# Supplementary material for: Molecular-level insights into the supramolecular gelation mechanism of urea derivative
Source: Nat Commun. 2025 Apr 22;16:3758. doi: 10.1038/s41467-025-59032-6 (PMC12015314; doi:10.1038/s41467-025-59032-6)
Supplement: Supplementary file 1 — Supplementary Information [file 41467_2025_59032_MOESM1_ESM.pdf]

Supplementary Information

Molecular-level insights into the supramolecular gelation mechanism of urea derivative

Shinya Kimura[1*], Kurea Adachi[1], Yoshiki Ishii[2], Tomoki Komiyama[1,3], Takuho Saito[4], Naofumi Nakayama[5], Masashi Yokoya[1], Hikaru Takaya,[6,7] Shiki Yagai[8,9*], Shinnosuke Kawai[3*], Takayuki Uchihashi[2,10*], Masamichi Yamanaka[1]

[1]Meiji Pharmaceutical University, 2-522-1 Noshio, Kiyose, Tokyo 204-8588, Japan.

[2]Department of Physics and Institute for Glyco-core Research (iGCORE), Nagoya University, Furo-cho, Chikusa-ku, Nagoya 464-8602, Japan.

[3]Department of Chemistry, Faculty of Science, Shizuoka University, 836 Ohya, Suruga-ku, Shizuoka 422-8529, Japan.

[4]Division of Advanced Science and Engineering, Graduate School of Science and Engineering, Chiba University, 1-33 Yayoi-cho, Inage-ku, Chiba 263-8522, Japan.

[5]CONFLEX Corporation, 3-23-17 Takanawa, Minato-ku, Tokyo 108-0074, Japan.

[6]Department of Life Science, Faculty of Life & Environmental Sciences, Teikyo University of Science, Main Build #15-05, 2-2-1 Senjyusakuragi, Adachi-ku, Tokyo 120-0045, Japan.

[7]Division of Advanced Molecular Science, Institute for Molecular Science, National Institute of Natural Science, 38 Aza-Saigo-Naka, Myodaiji, Okazaki, Aichi 444-8585, Japan.

[8]Department of Applied Chemistry and Biotechnology, Graduate School of Engineering, Chiba University, 1-33 Yayoi-cho, Inage-ku, Chiba 263-8522, Japan.

[9]Institute for Advanced Academic Research (IAAR), Chiba University, 1-33 Yayoi-cho, Inage-ku, Chiba 263-8522, Japan.

[10]Exploratory Research Center on Life and Living Systems (ExCELLS), National Institutes of Natural Sciences, 5-1 Myodaiji, Okazaki, Aichi 444-8787, Japan.

*E-mail: s-kimura@my-pharm.ac.jp; yagai@faculty.chiba-u.jp; sskawai@shizuoka.ac.jp; uchihast@d.phys.nagoya-u.ac.jp

This PDF file includes:

Supplementary Methods

Supplementary Figures and Tables

Supplementary References

Supplementary Methods

Synthesis of UC13: UC13 was synthesized according to our previous report.[1] All starting materials and reagents were purchased from commercial suppliers and used without further purification.

A mixture of 2-benzylaniline (3.30 g, 18.0 mmol), 4-nitrophenyl chloroformate (3.63 g, 18.0 mmol), and pyridine (1.45 mL, 18.0 mmol) in 1,2-dichloroethane (180 mL) was stirred at 70 °C for 14 h under argon atmosphere. The reaction mixture was cooled to room temperature and 15% NaOH solution was added. The organic layer was washed with H_2O (3 times) and brine, and dried over Na_2SO_4. The solvent was removed under reduced pressure, and the crude product was purified by reprecipitation (CH_2Cl_2/*n*-hexane). The desired 4-nitrophenyl (2-benzylphenyl)carbamate was obtained as a white solid (5.70 g, 91%).

n-Tridecylamine (3.02 mL, 12.4 mmol) was added dropwise to a solution of the above-mentioned carbamate (4.32 g, 12.4 mmol) and Et_3N (1.72 mL, 12.4 mmol) in CH_2Cl_2 (125 mL). The reaction mixture was stirred at room temperature for 17 h under argon atmosphere. The solvent was removed under reduced pressure, and the crude product was purified by recrystallization (CH_3CN). The desired product **UC13** was obtained as a white solid (4.41 g, 94%).

mp: 119-120 °C; ^{1}H NMR (400 MHz, DMSO-*d*$_6$): δ 7.76–7.71 (m, 2H), 7.26 (dd, *J* = 7.6, 7.6 Hz, 2H), 7.21–7.14 (m, 3H), 7.11 (dd, *J* = 7.6, 7.6 Hz, 1H), 6.99 (d, *J* = 6.8 Hz, 1H), 6.90 (dd, *J* = 7.3, 7.3 Hz, 1H), 6.45 (t, *J* = 5.8 Hz, 1H), 3.90 (s, 2H), 3.04 (dq, *J* = 5.8, 7.1 Hz, 2H), 1.43–1.35 (m, 2H), 1.32–1.18 (m, 20H), 0.84 (t, *J* = 6.8 Hz, 3H); ^{13}C NMR (125 MHz, $CDCl_3$): δ 156.6, 139.5, 136.1, 135.8, 131.3, 128.9, 128.7, 128.0, 126.64, 126.56, 126.5, 40.7, 38.2, 32.06, 30.12, 29.82, 29.79, 29.72, 29.68, 29.5, 29.4, 27.0, 22.8, 14.3; **HRMS** (ESI, *m/z*) $[M+Na]^+$ calcd. for $C_{27}H_{40}N_2NaO$, 431.3033; found, 431.3027.

Gelation experiments: A mixture of **UC13** and DMSO (500 μL) in a glass vial was heated on a hot plate (150 °C) until dissolved. The obtained solution was gradually cooled to ambient temperature. Gel formation was evaluated by the inverted tube test. A mixture remaining at the top of an inverted glass vial was defined as a gel.

A mixture of **UC13** and EMI-Tf_2N (500 μL) in a glass vial was heated on a hot plate (200 °C) until dissolved. The obtained solution was gradually cooled to ambient temperature. Gel formation was evaluated by the inverted tube test. A mixture remaining at the top of an inverted glass vial was defined as a gel.

Time-course Analysis of Gelation: A mixture of **UC13** and EMI-Tf_2N (500 μL) in a glass vial was heated on a hot plate (200 °C) until dissolved. The obtained solution was gradually cooled to ambient temperature. Ten vials of the same solution were prepared for each concentration. The change in turbidity in a vial was monitored by recording a digital movie by iPad Air (5th generation). Frames were extracted every one minute from the movie file, and image analysis to evaluate the turbidity was performed. In the first frame, two rectangular regions for each vial were assigned, one enclosing the vial and the other designating the solution region. In the subsequent frames, the vial rectangles were re-assigned through template-matching with the image in the previous frame by evaluating the zero-mean normalized cross-correlation. The position of the solution rectangle is accordingly re-assigned by keeping the relative position from the vial rectangle. Turbidity was evaluated by calculating the average brightness of the pixels contained in the solution rectangle. To minimize the effect of the room illumination, the average brightness in the upper half of the vial rectangle was subtracted as background. Then, the obtained turbidity values as a function of time were fitted to the following analytic formula:

$$f(t) = b + \frac{a}{1 + \exp\left(-k(t - t_{\mathrm{c}})\right)},$$

where t is time, and the parameters a, b, t_{c}, and k were estimated by least-squares fitting. In this formula, the turbidity values before and after the gelation are given by $f(-\infty) = b$ and $f(+\infty) = a + b.$ Then the gelation start time t_{start} was defined as $t_{\mathrm{start}} = t_{\mathrm{c}} - 2a/k.$ This is the time at which the line tangent to $y = f(t)$ at $t = t_{\mathrm{c}}$ cuts the horizontal line $y = b.$ Similarly, the gelation completion time t_{comp} was defined as $t_{\mathrm{comp}} = t_{\mathrm{c}} + 2a/k,$ the time at which the same tangent line cuts $y = a + b.$

High-speed atomic force microscopy (HS-AFM): HS-AFM experiments were conducted with a laboratory-built instrument, utilizing an Olympus microcantilever (BL-AC7, Olympus) operated in the tapping mode.[2] The microcantilever, with a nominal spring constant of 0.2 N/m and a resonant frequency of approximately 600 kHz in solution, does not come equipped with a sharp tip. To obtain high-quality AFM images, a carbon pillar was deposited at the end of the cantilever via electron-beam deposition in carbon-containing gas evaporated from ferrocene. This carbon pillar was then honed into a sharp probe with an approximate radius of 4 nm through plasma etching with argon gas.

HOPG was chosen as the observation substrate, given that the **UC13** fibers did not adsorb onto the normally used mica surface for HS-AFM observations. HOPG was machined into a circular disc with a diameter of 2 mm and affixed to a glass stage for HS-AFM observations using acrylic adhesive. A clean surface was obtained by cleavage with Scotch tape immediately before the measurement. The substrate was then immersed in DMSO or EMI-Tf_2N. The **UC13** powder was first dispersed in the solvent and heated to 200 °C, using a hot stirrer to dissolve it. The gelation of **UC13** was then monitored by adding the appropriate amount of the dissolved **UC13** to the observation solvent (the ionic liquid) during the HS-AFM observation. The total volume of the solvent used was 70 μL.

In the HS-AFM experiments, we obtained a phase image as well as a topographic image. The phase image corresponds to the change in the phase of the cantilever oscillation with reference to the cantilever excitation signal, which is detected using a lock-in amplifier (HF2LI, Zurich Instruments). Phase imaging often yields better contrast and higher resolution than topographic images in fiber imaging (Supplementary Fig. 1). Therefore, the discussion in this article has been based on phase images, unless stated otherwise.

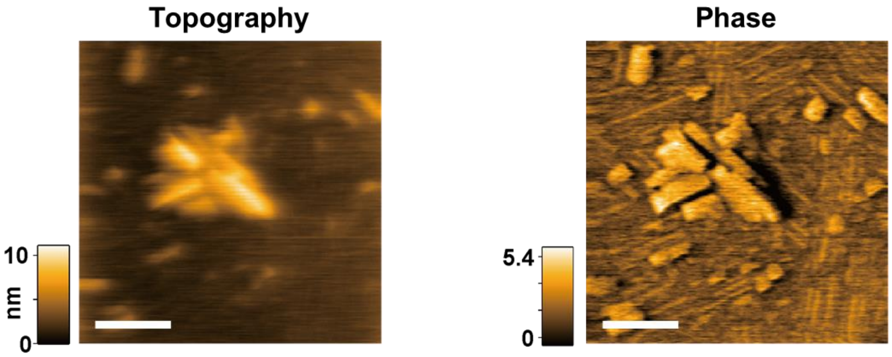

Supplementary Figure 1. Topographic image and phase image of fibers in the third step. Representative images from numerous experiments showing similar results are presented. Scale bars: 100 nm.

Dynamic light scattering (DLS): DLS measurements were performed using a Malvern Panalytical Instruments Zetasizer Nano S device equipped with a 4.0 mW He-Ne laser (λ = 633 nm) using a non-invasive back-scattering (NIBS) technology. The scattering angle was set at 173°. The values of viscosity of 45.8 mPa·s and refractive index of 1.4230 of EMI-Tf_2N at 25 °C were assumed.[3,4] To obtain the DLS size distributions, the non-negative least squares (NNLS) regularization algorithm was applied to analyze the DLS autocorrelation functions. The DLS data were processed using Zetasizer Software 7.11. Samples for DLS measurements were prepared by vigorously heating solid samples of **UC13** in EMI-Tf_2N until completely dissolved, and then the resulting solution was cooled to 25 °C. The solution was transferred to a quartz cuvette for DLS measurements.

Conformational search for UC13: The conformations around the four bonds of **UC13** as shown in Supplementary Fig. 2 were searched under the assumption that the two NH bonds of the urea group were in the same direction and that the conformation of the tridecyl chain was all *trans* in terms of self-assembly. The initial conformational search was performed using CONFLEX9.C[5-7] program with MMFF94s[8-14] force field. The conformational isomers thus obtained were further optimized using the density functional theory (DFT) method at the B3LYP/6-311+G(2d,p) level performed using the Gaussian 16 program.[15]

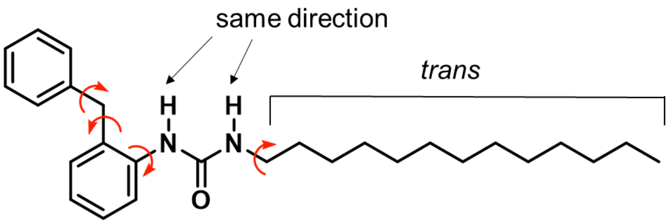

Supplementary Figure 2. Structural assumptions for the exploration of **UC13** conformations. The bonds selected for conformational search are indicated by red curved arrows, and the two (O=)C-N(-H) bonds in the urea group and the eleven C-C bonds in the alkyl chain were excluded from the conformational search, based on self-assembly considerations.

Interatomic distance between UC13 molecules in the dimer: For the purpose of clarifying the intermolecular structure between **UC13** molecules, geometry optimization of the **UC13** dimer was performed at the B3LYP-D3/6-311+G(2d,p) level[16] using the Gaussian 16 program (Supplementary Fig. 3).[15] The initial geometry of the dimer was constructed based on the monomer in conformation No. 4 (Supplementary Fig. 5); one molecule was set to the original coordinates, and the other was shifted in the y and z directions by -4 Å.

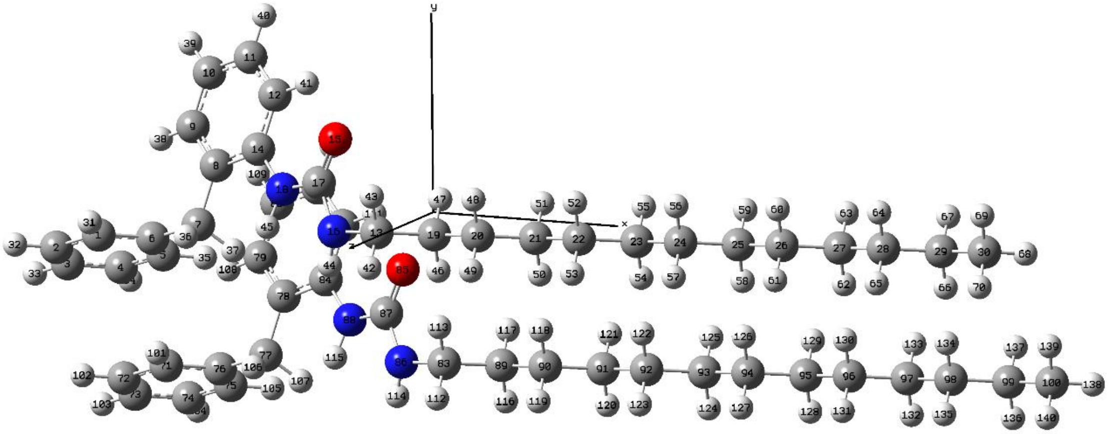

Supplementary Figure 3. Initial geometry of the **UC13** dimer used for structure optimization. The dimer was constructed from the **UC13** monomer in conformation No. 4 shown in Supplementary Fig. 5, with one molecule shifted by −4 Å along the y and z axes. Colour code: carbon, grey; oxygen, red; nitrogen, blue; hydrogen, white.

Single-crystal X-ray crystallographic analyses of UCCy: Single crystals of **UCCy** suitable for X-ray diffraction studies were analyzed by using synchrotron X-ray radiation at SPring-8 (BL40XU) as described in Figure 2d and Supplementary Fig. 6. The molecular structure of **UCCy** was solved by direct methods and refined by the full-matrix least squares method. The positions of all non-hydrogen atoms were found from difference Fourier electron density maps and refined anisotropically. All calculations were performed using the Rigaku Crystal Structure crystallographic software packages or Yadokari-XG (Wakita, Nemoto et al., 2009). These crystallographic data have been deposited with Cambridge Crystallographic Data Centre: Deposition number CCDC-2321499, for compound **UCCy**. Copies of the data can be obtained free of charge via http://www.ccdc.cam.ac.uk/conts/retrieving.html (or from the Cambridge Crystallographic Data Centre, 12, Union Road, Cambridge, CB2 1EZ, UK; Fax: +44 1223 336033; e-mail: deposit@ccdc.cam.ac.uk).

XRD Analysis: The gel samples for SAXS analysis were prepared in a quartz glass capillary tube (2.0 mmϕ) and measured at BL19B2 beamline with a q-range from 0.047 to 33.121 nm^{-1} under the beamline standard conditions[17] as shown in Supplementary Fig. 7a. The WAXS measurements of gel samples were performed at BL02B2 beamline in SPring-8 with a 2θ range from 1.0 to 83.0 deg using a Lindeman glass capillary tube (0.2 mm) under the beamline standard condition[18] as shown in Supplementary Fig. 7b.

Numerical simulations of fiber growth by the *block-stacking model*: The supramolecular fiber is modeled as a bundle of fibers consisting of monomers represented as blocks in Figure 7a. Here the "monomer" does not necessarily mean a single molecule. The molecules may form some small aggregates in solution, and these aggregates act as a unit block for the formation of larger fibers. In this model, such a small aggregate acting as a building block for the fiber is represented as one block in Figure 7. To keep the model simple, we express the size polydispersity of the aggregates in solution by assuming that the molecules exist in the solution in the form of either a single block ("monomer," light orange blocks in Figure 7a) or two blocks glued to each other ("dimer," dark red blocks in Figure 7a).

In the simulation, the blocks are arranged into a 4×4 grid in the horizontal direction, and the fiber growth is assumed to occur via binding of monomers or dimers in the vertical direction (Figure 7a). The rates of association of a new block upon one site is set to be $k_\mathrm{s} = 2.5 \times 10^{-1}\,\mathrm{s}^{-1}$ if there is at least one horizontal interaction available for the newly stacked block, and $k_\mathrm{t} = 2.5 \times 10^{-4}\,\mathrm{s}^{-1} = 10^{-3} k_\mathrm{s}$ if there is no horizontal interaction. The rate constant k_s is set 1000 times larger than k_t reflecting the assumption that the adsorbed block is stabilized through horizontal interactions with neighboring blocks. The distribution of monomers and dimers in solution is assumed to be $p_1 = 0.9$ and $p_2 = 0.1$, where p_1 and p_2 are the proportion of monomers and dimers respectively.

The numerical simulation is performed by the Gillespie method.[5,19–21] At each step, random numbers are generated to decide when and at which site the next block is adsorbed. The rate of the adsorption for each of the 4×4 sites is either k_s or k_t as described above. Summing the rates for all the sites gives the probability distribution for the time when the next adsorption occurs, whence the time is randomly picked according to this distribution. Then the site on which the adsorption occurs is decided by another random sampling according to the ratios of the rate constants for all the sites. Finally, whether a monomer or a dimer is adsorbed is decided randomly by the probability distribution (p_1, p_2) given above. This procedure was repeated for 300 steps to generate the growth curve of Figure 7b. The elongation distance of the fiber is evaluated at each step as the average height of the 4×4 sites. The height of one block is assumed to be $4\,\mathrm{nm}$ to semi-quantitatively reproduce the experimental result. The results of this simulation are shown in Supplementary Figs. 11 and 12.

Probability Distribution of the Nucleation Time: We adopt here a simple model of nucleation where s monomers (M) undergo an association reaction to form a nucleus (N) that triggers the fiber growth:

$$s\mathrm{M} \rightarrow \mathrm{N}. \tag{S1}$$

The rate of the reaction (S1) is given by $k[\mathrm{M}]^s$ with rate constant k. The reaction (S1) can be a composite of multiple steps each of which corresponds to the adsorption of a single monomer to an existing oligomer. However, as the formation of the s-mer (N) is assumed to be the rate-determining step, the pre-equilibrium scheme yields the rate law of $k[\mathrm{M}]^s$ to good approximation.

Let $P_1(t)$ be the probability that at least one nucleus has been formed in the sample solution at time t. It obeys the following differential equation:

$$\frac{\mathrm{d}}{\mathrm{d}t} P_1(t) = kV[\mathrm{M}]^s \big(1 - P_1(t)\big), \tag{S2}$$

where V is the volume of the solution. As we start with a monomer solution at time zero, that is, $P_1(0) = 0$, Eq. (S2) can readily be solved as follows.

$$P_1(t) = 1 - \exp(-kV[\mathrm{M}]^s t). \tag{S3}$$

The probability that a nucleus is formed between t and $t + \mathrm{d}t$ is then given by

$$P_1(t + \mathrm{d}t) - P_1(t) = \mathrm{d}P_1(t) = kV[\mathrm{M}]^s \exp(-kV[\mathrm{M}]^s t)\ \mathrm{d}t. \tag{S4}$$

This gives the probability density function $P(t_{\mathrm{nuc}})$ for the nucleation time t_{nuc}.

$$P(t_{\mathrm{nuc}}) = kV[\mathrm{M}]^s \exp(-kV[\mathrm{M}]^s t) \tag{S5}$$

Eq. (S5) has the form of the exponential distribution. The expectation $\langle t_{\mathrm{nuc}} \rangle$ and standard deviation $\mathrm{SD}[t_{\mathrm{nuc}}]$ of t_{nuc} are readily calculated from Eq. (S5).

$$\langle t_{\mathrm{nuc}} \rangle = \int_0^{+\infty} t_{\mathrm{nuc}} P(t_{\mathrm{nuc}})\, \mathrm{d}t_{\mathrm{nuc}} = (kV[\mathrm{M}]^s)^{-1}. \tag{S6}$$

$$\langle {t_{\mathrm{nuc}}}^2 \rangle = \int_0^{+\infty} {t_{\mathrm{nuc}}}^2 P(t_{\mathrm{nuc}})\, \mathrm{d}t_{\mathrm{nuc}} = 2(kV[\mathrm{M}]^s)^{-2}. \tag{S7}$$

$$\mathrm{SD}[t_{\mathrm{nuc}}] = (\langle {t_{\mathrm{nuc}}}^2 \rangle - \langle t_{\mathrm{nuc}} \rangle^2)^{1/2} = (2(kV[\mathrm{M}]^s)^{-2} - (kV[\mathrm{M}]^s)^{-2})^{1/2} \tag{S8}$$

$$= (kV[\mathrm{M}]^s)^{-1}$$

As a general property of the exponential distribution, the standard deviation is equal to the expectation: $\mathrm{SD}[t_{\mathrm{nuc}}] = \langle t_{\mathrm{nuc}} \rangle$.

Supplementary Figures and Tables

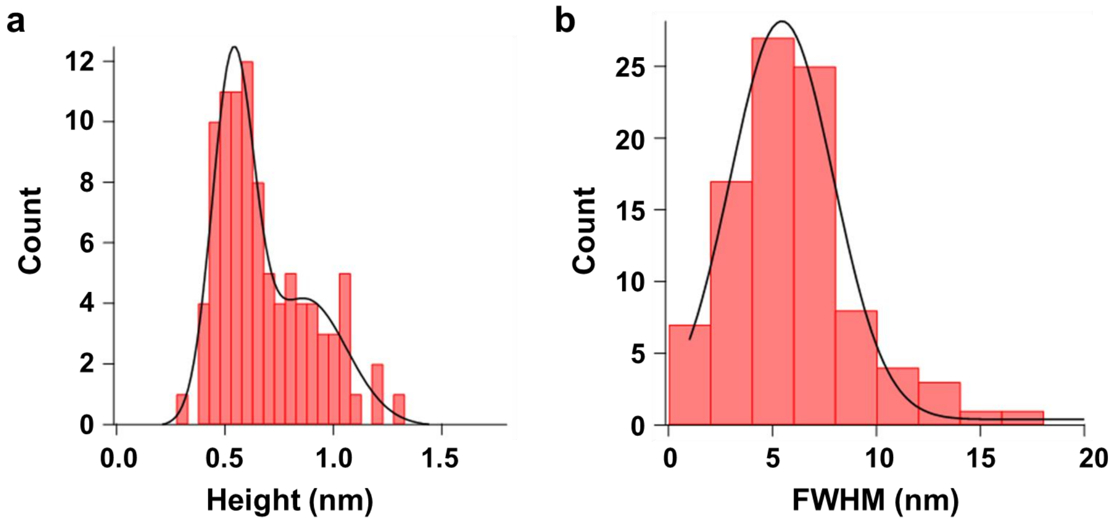

Supplementary Figure 4. Height and thickness of fibrils formed from **UC13**(30 mM) in DMSO. **a**, Histogram of the height of the fibrils (*n* = 94). The black curve indicates the Gaussian fitting with the center values of 0.5 nm (standard deviation: 0.2 nm) and 0.9 nm (deviation: 0.5 nm). These two peaks indicate the presence of **UC13** layers. **b**, Histogram of the full width at half maximum (FWHM) of the fibrils. The black curve indicates the Gaussian fitting with the center value of 5.5 nm (deviation: 3.5 nm). Source data of the graphs are provided as a Source Data file.

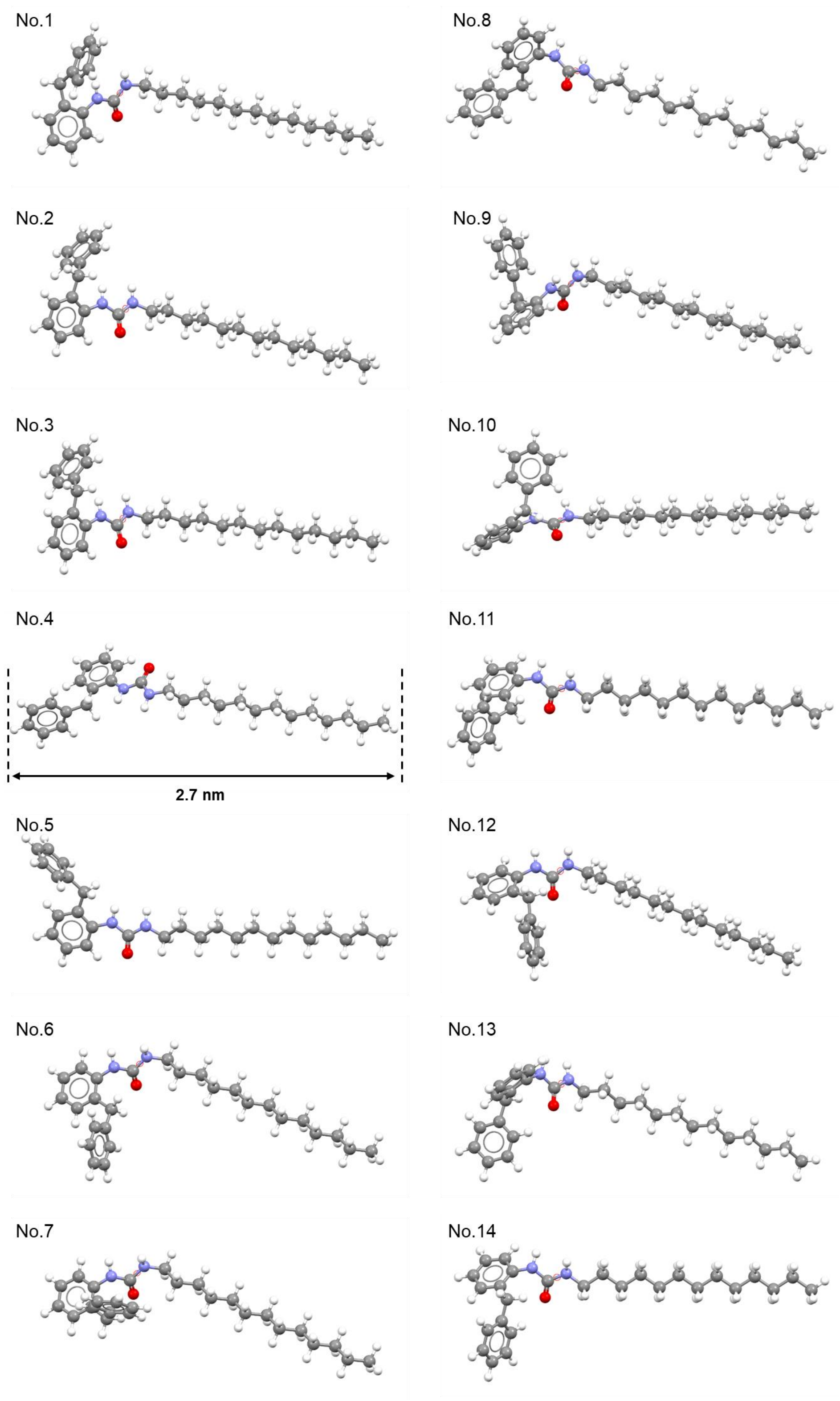

Supplementary Figure 5. Representative conformations of **UC13** obtained from DFT

calculations. Colour code: carbon, grey; oxygen, red; nitrogen, blue; hydrogen, white. The relative energies and distances between the farthest protons in the 14 conformational isomers are listed in Supplementary Table 2. Since the hydrogen-bonding site of the urea group of conformers No. 1–3 is partially covered with a 2-benzylphenyl group, conformation No. 4 was adopted in Figure 4a.

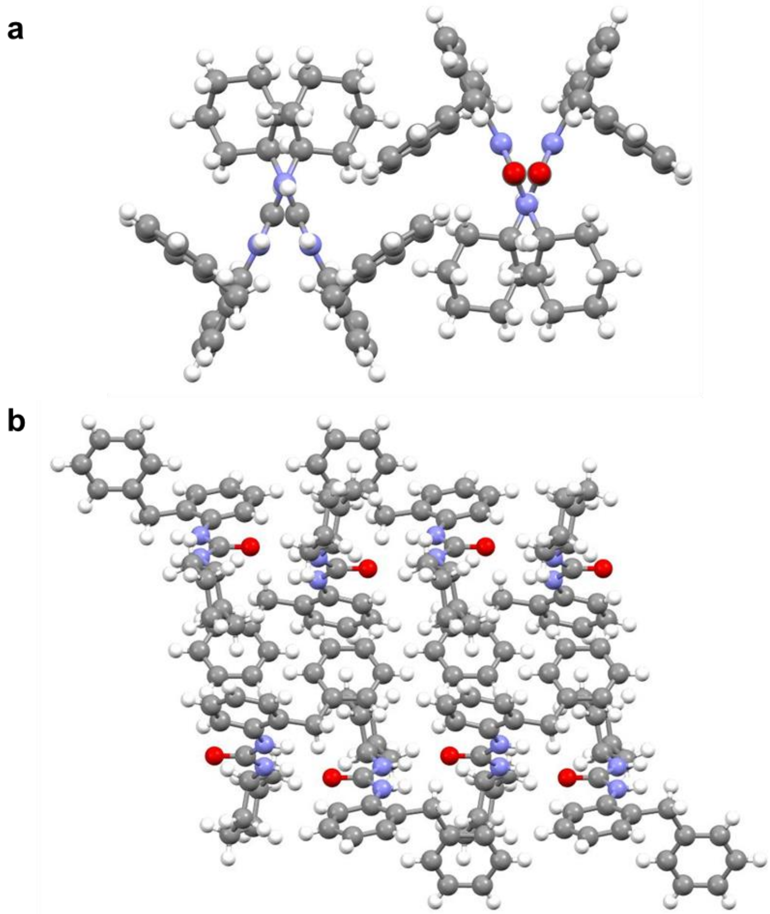

Supplementary Figure 6. Refined crystal structure of **UCCy** in the space group P-1 at 90 K. Mercury ball-and-stick plots of the complex are shown, viewed along (a) the *a*-axis and (b) the *c*-axis. These views show the molecular columns in which the **UCCy** molecules are conjugated through hydrogen-bonding linkages. Colour code: carbon, grey; oxygen, red; nitrogen, blue; hydrogen, white.

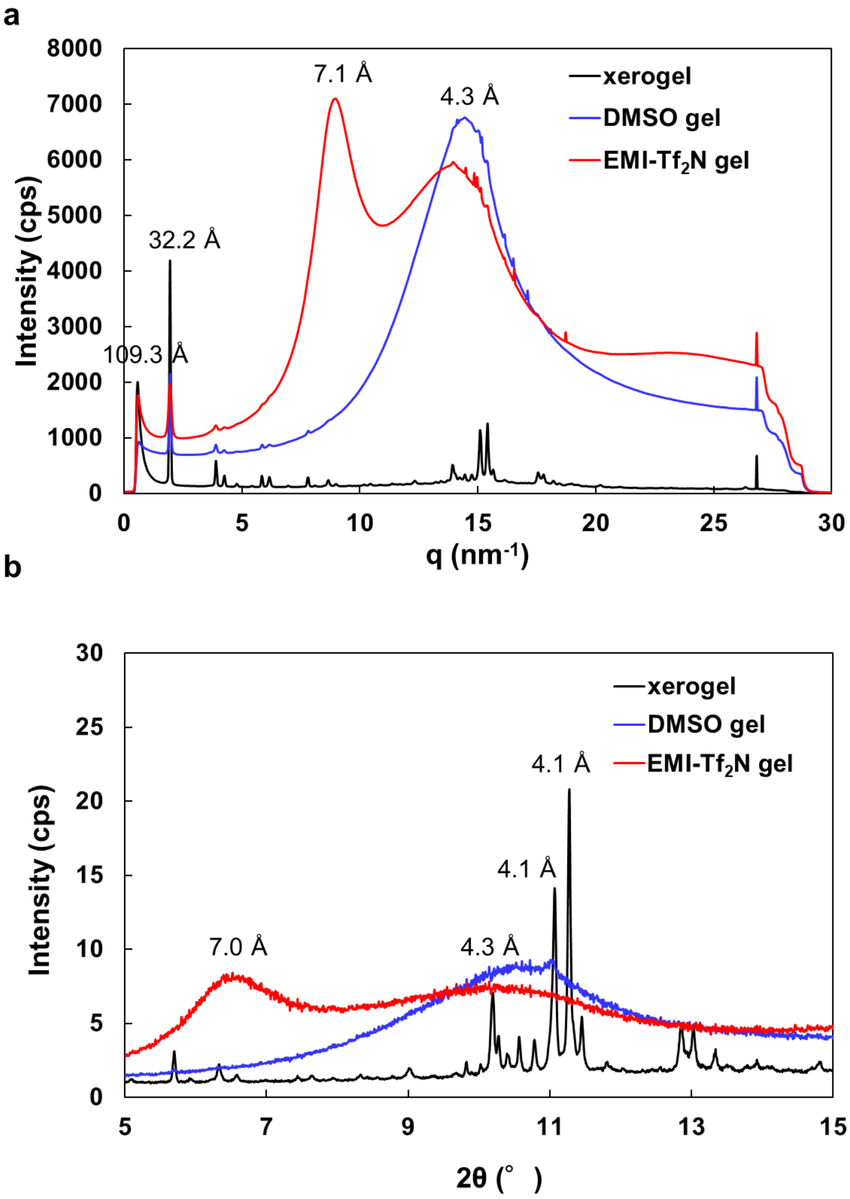

Supplementary Figure 7. X-ray scattering patterns of **UC13**. **a**, SAXS pattern of **UC13**-Xerogel (black), **UC13**-DMSO gel (66.5 mM, blue), and **UC13**-EMI-Tf_2N gel (50.0 mM, red). **b**, Enlarged WAXS pattern of **UC13**-Xerogel (black), **UC13**-DMSO gel (66.5 mM, blue), and **UC13**-EMI-Tf_2N gel (50.0 mM, red). A peak at 109.3 Å, corresponding to 0.6 nm^{-1} in Supplementary Fig. 7a, was also observed in the blank, in which only the quartz glass capillary was measured. The distance of 32.2 Å, corresponding to the 1.9 nm^{-1} in Supplementary Fig. 7a, is slightly larger than the molecular length of **UC13** (2.7 nm). For instance, this distance may correspond to a self-assembly in parallel manner aggregates (Figure 6a). Source data of the graphs are provided as a Source Data file.

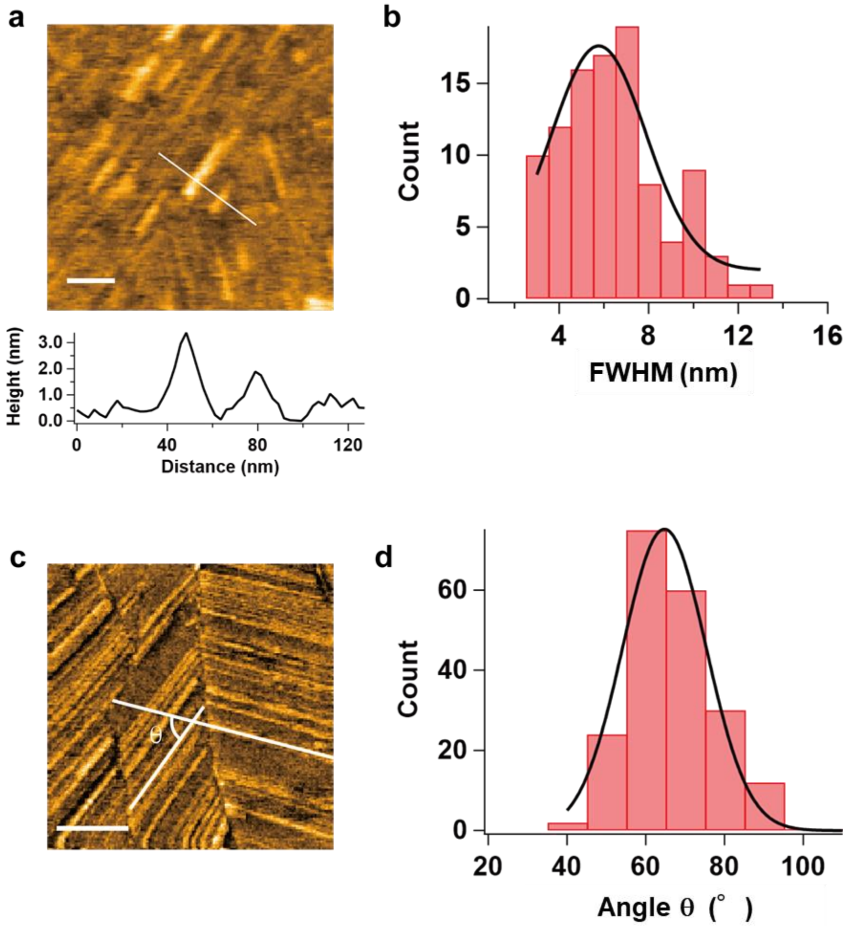

Supplementary Figure 8. Thickness and orientation of fibrils formed in the second step. **a**, (upper) A topographic HS-AFM image of the fibrils in the second step (2.0 mM **UC13**) and (lower) the cross section marked along the white line on the image. Scale bar: 50 nm. A representative image from more than five independent experiments with similar results is shown. Source data of the line profile are provided as a Source Data file. **b**, Histogram of the full width at half maximum (FWHM) of the fibrils (*n* = 100). The black curve indicates the Gaussian fitting with the center value of 5.8 nm (deviation: 3.0 nm). Source data of the histogram are provided as a Source Data file. **c**, Phase-contrast HS-AFM image showing anisotropic adsorption of intermediate fibrils onto the HOPG surface. Scale bar: 100 nm. **d**, Histogram of angles between the fibrils, as defined by the angle drawn on the image (*n* = 203) with the center value of 64.7 degrees and the deviation of 15.0 degrees. A representative image from more than five independent experiments with similar results is shown. Source data of the histogram are provided as a Source Data file.

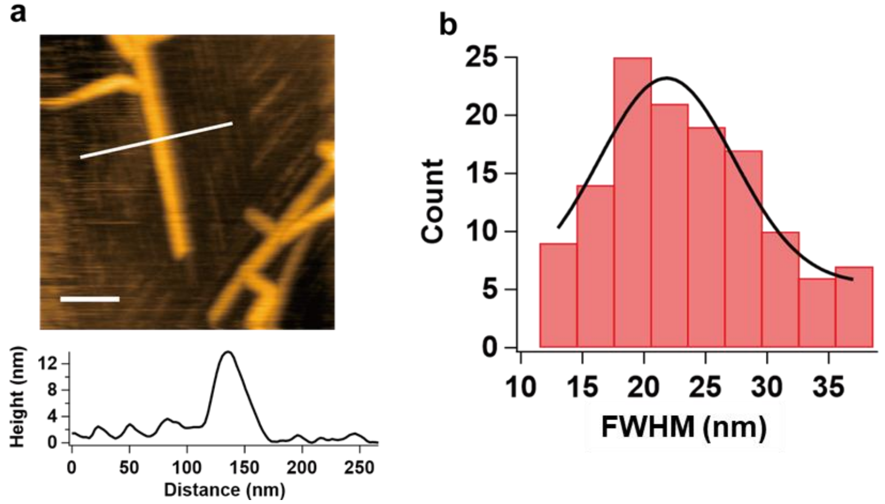

Supplementary Figure 9. Thickness of the fiber in the third step. **a**, (Upper) Topographic HS-AFM image of the fibers in the third step (3.0 mM **UC13**); (Lower) Cross section marked along the white line on the image. Scale bar: 100 nm. A representative image from more than five independent experiments with similar results is shown. **b**, Histogram of full width at half maximum (FWHM) of the fibers (n = 128). The black curve represents the Gaussian fitting with the center value of 21.8 nm (deviation: 7.7 nm). Source data of the histogram are provided as a Source Data file.

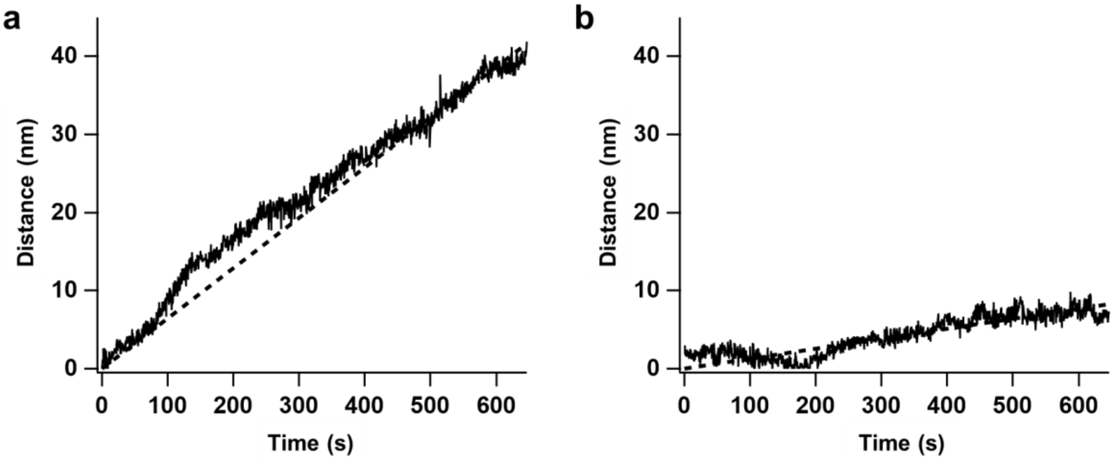

Supplementary Figure 10. Anisotropic self-assembly of **UC13**. **a**, Growth distance of the L-end as a function of time. **b**, Growth distance of the R-end as a function of time. Representative data from more than four different fibers with similar results are shown. Source data of the graphs are provided as a Source Data file.

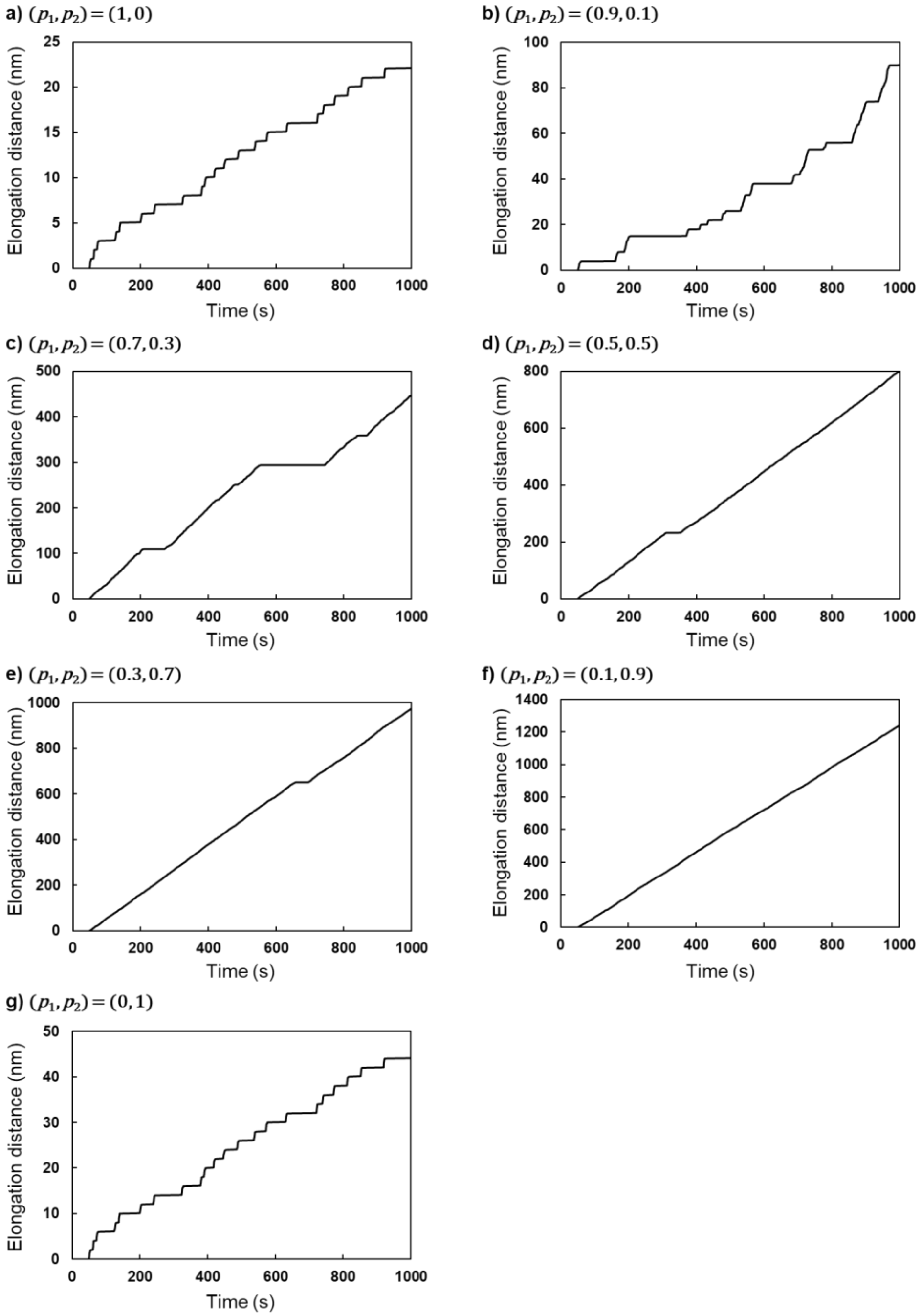

Supplementary Figure 11 Simulation of the block-stacking model. The proportion p_2 of dimer is assumed to be (a) 0, (b) 0.1, (c) 0.3, (d) 0.5, (e) 0.7, (f) 0.9, (g) 1. Here, the binding rate of binding units stabilized by lateral interactions is assumed to be 1,000 times the binding rate in the absence of such interactions. This simulation suggests that intermittent growth with repeated elongation and pause phases occurs in most cases. Therefore, such a growth manner is likely to be a common phenomenon. Source data of the graphs are provided as a Source Data file.

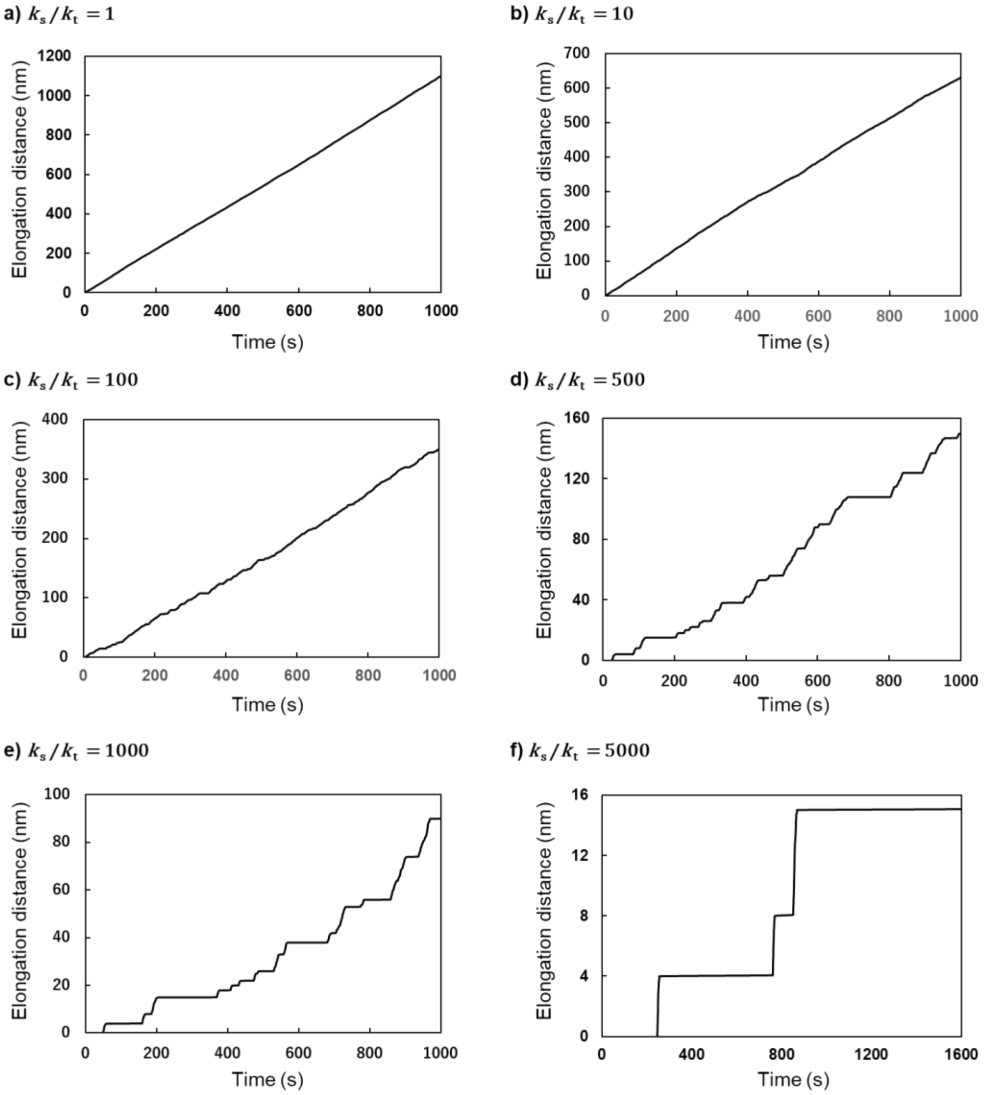

Supplementary Figure 12. Simulation of the block-stacking model. The binding rate of binding units stabilized by lateral interactions is assumed to be (a) 1, (b) 10, (c) 100, (d) 500, (e) 1,000, (f) 5,000 times the binding rate observed in the absence of such interactions. Here, the proportion of dimer is assumed to be 0.1. This simulation suggests that a stabilizing effect of 500 times or more would lead to intermittent growth with repeated elongation and pause phases. Considering the Boltzmann distribution, the fiber should benefit from a lateral interaction of approximately 15 kJ/mol, which is the energy that can be acquired by π–π stacking and van der Waals interactions. This growth manner may commonly occur in bundling fibers. Source data of the graphs are provided as a Source Data file.

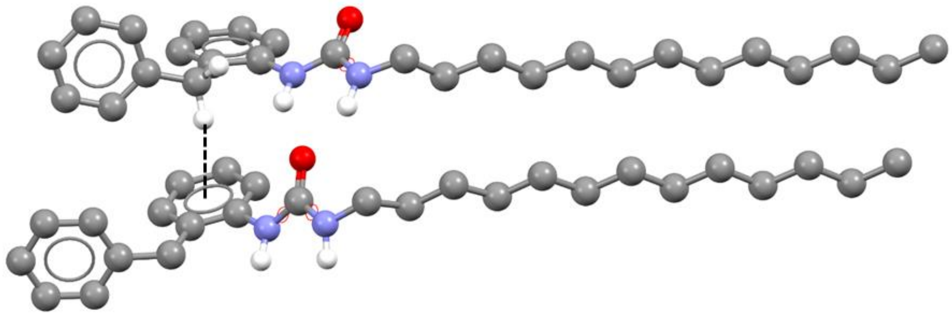

Supplementary Figure 13. Optimized geometry of the parallel dimer of **UC13** obtained from DFT calculations. Colour code: carbon, grey; oxygen, red; nitrogen, blue; hydrogen, white. The initial geometry used for structure optimization is shown in Supplementary Fig. 3. A CH-π interaction is suggested between the monomers, as indicated by the dotted line. The closest (C-)H···C(Ph) distance is 2.86 Å.

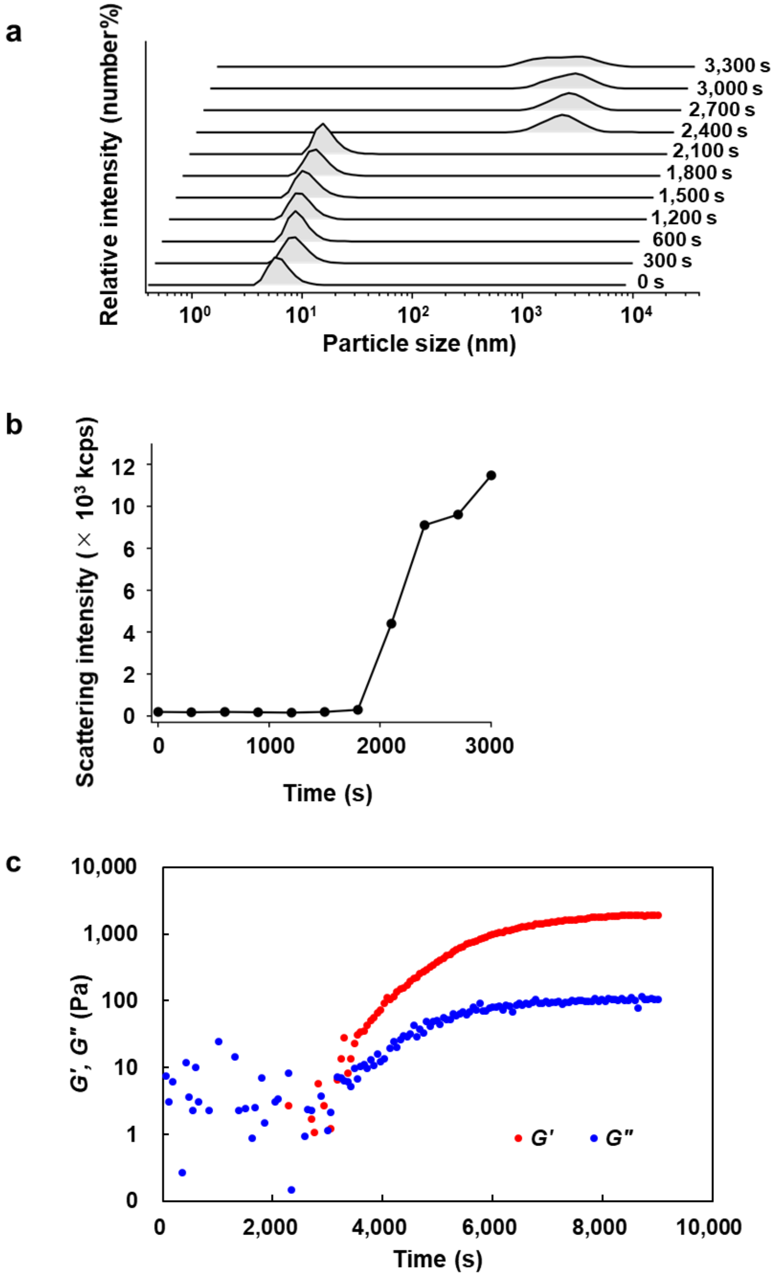

Supplementary Figure 14. Time course of the gelation process. **a**, DLS time course of the particle size in the 3.0 mM **UC13** in EMI-Tf_2N every 5 min. **b**, DLS time course of signal intensity in the 3.0 mM **UC13** in EMI-Tf_2N. Source data of the graphs are provided as a Source Data file. **c**, Time course of the storage modulus (*G′*) and loss modulus (*G″*) for the gelation of the 2.5 mM **UC13** in EMI-Tf_2N every min. Source data of the graphs are provided as a Source Data file.

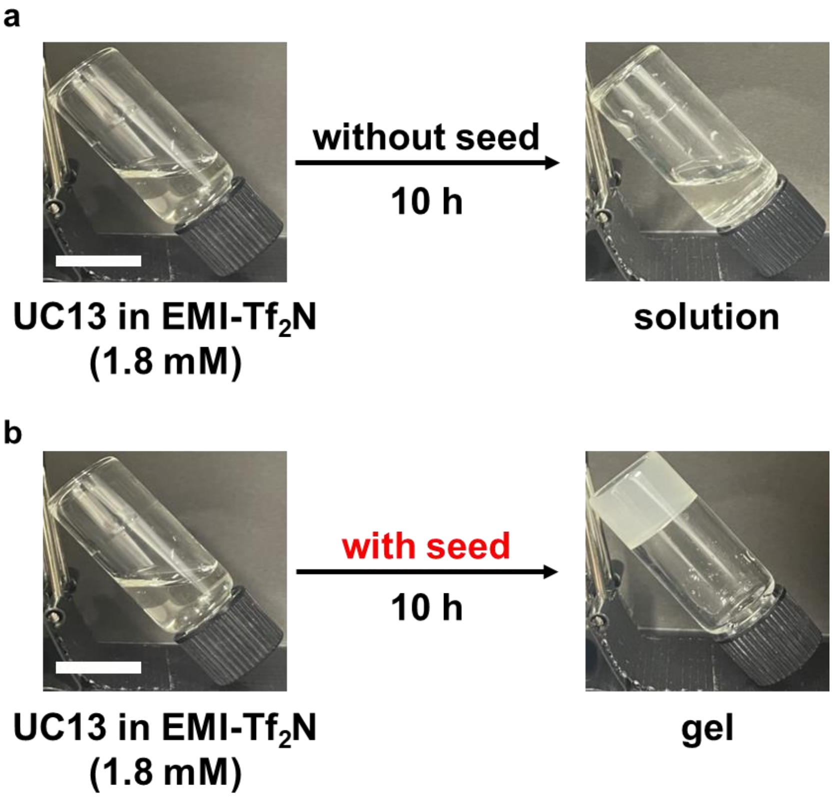

Supplementary Figure 15. Photographs of acceleration of gelation with addition of seeds. (a) Without seed. (b) With a trace amount of seed (1 mol%). Scale bars: 10 mm.

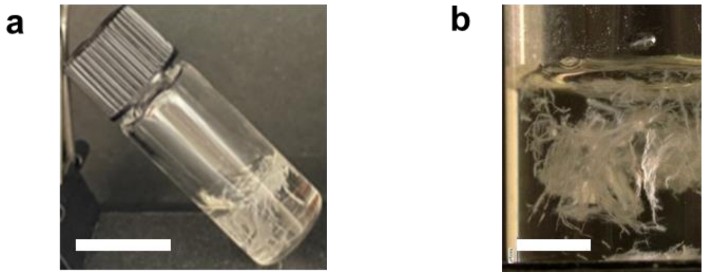

Supplementary Figure 16. Photographs of the suspension of fibrous macroscopic aggregates formed in the 1.5 mM **UC13** in EMI-Tf_2N. (a) Whole view. Scale bar: 10 mm. (b) Enlarged view. Scale bar: 2.5 mm.

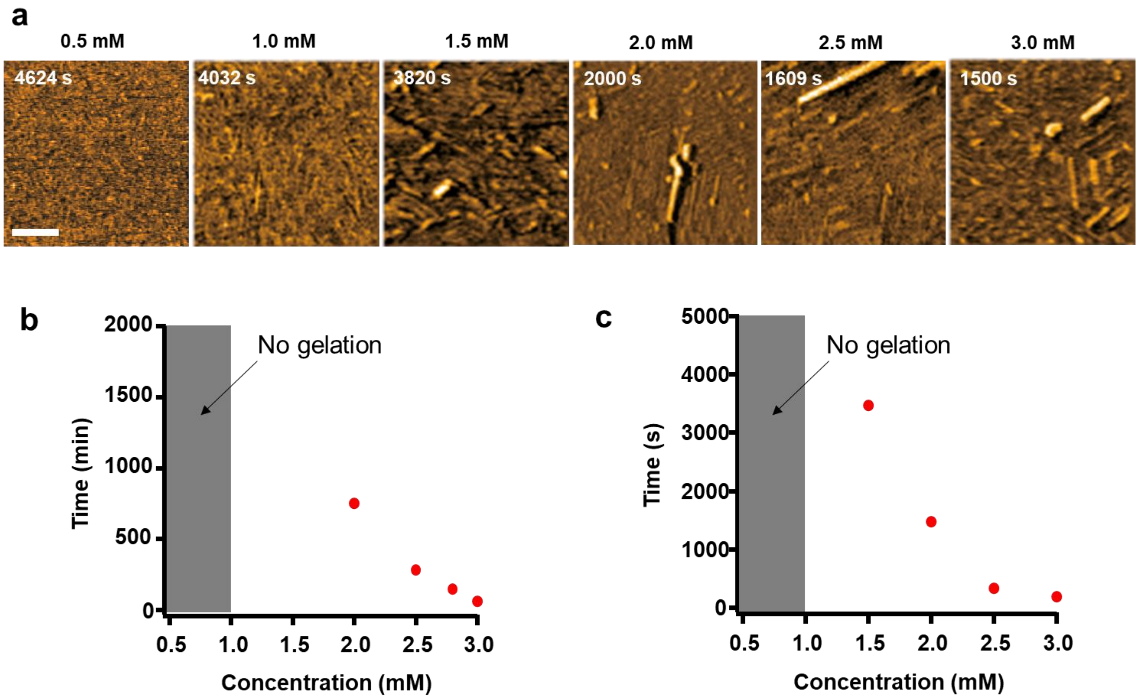

Supplementary Figure 17. Relationship between the growth rate of the fiber/gelation time and **UC13** concentration. **a**, AFM images obtained at various **UC13** concentrations of 0.5, 1.0, 1.5, 2.0, 2.5, and 3.0 mM. Representative images from more than three independent experiments are shown. **b**, Concentration-dependence of **UC13** on gelation time. **c**, Effect of **UC13** concentration on the time required for fiber formation in the third step. Representative data from more than three independent experiments are shown. Source data of the graphs are provided as a Source Data file.

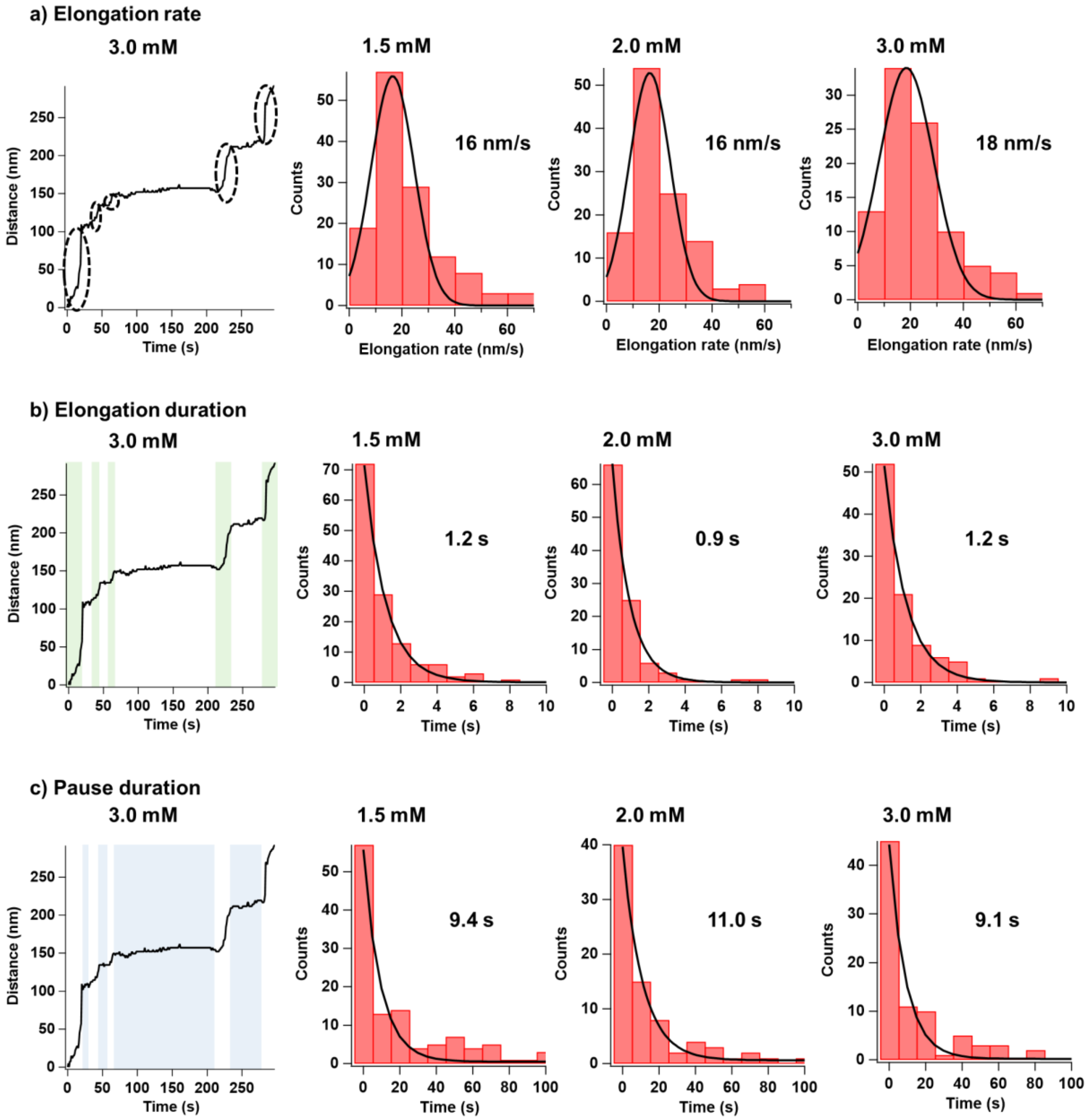

Supplementary Figure 18. Growth rate and duration of the third step fiber. The graphs on the left in Supplementary Fig. 18a–c shows the growth rate of typical fibers (3.0 mM). In Supplementary Fig. 18a, the growth rate of the area circled by the dotted line was analyzed. In Supplementary Fig. 18b, the times of the areas in the green square were measured. In Supplementary Fig. 18c, the times of the areas in the blue square were measured. **a**, Typical fiber elongation over time (left, 3.0 mM); Elongation rates in the area marked by the dotted lines on the distance vs time plots on the left (center and right). The black curve shows the Gaussian fitting, and the elongation rate was calculated from the median value. The elongation rates were 16, 16, 18 nm/s for the **UC13** concentrations of 1.5 (n = 132), 2.0 (n = 116), and 3.0 (n = 99) mM, respectively. **b**, Typical fiber elongation over time (left, 3.0 mM); Elongation durations highlighted by the green areas on the distance vs time plots on the left (center and

right). The black curves show a single exponential function fitting. The elongation durations were 1.2, 0.9, and 1.2 s at 1.5 (*n* = 132), 2.0 (*n* = 104), and 3.0 mM (*n* = 96), respectively. **c**, Typical fiber elongation over time (left, 3.0 mM); Pause durations highlighted by the blue areas on the distance vs time plots on the left (center and right). The black curves show a single exponential function fitting. Pause durations were 9.4, 11.0, and 9.1 s at 1.5 (*n* = 133), 2.0 (*n* = 86), and 3.0 mM (*n* = 85), respectively. Representative analysis was performed on one fiber out of many fibers that showed similar results. Source data of the histograms are provided as a Source Data file.

Supplementary Table 1. Gelation time of the 30 mM **UC13** in DMSO.*

Run	1	2	3	4	5	6	7	8	9	10
Gelation time (days)	29	4	14	29	32	17	16	28	16	5

Run	11	12	13	14	15	16	17	18	19	20
Gelation time (days)	27	25	33	16	27	27	14	30	16	27

*All runs were performed under the same conditions.

Supplementary Table 2. Properties of the conformational isomers of **UC13**.

No.[a)]	Relative Energy (kcal/mol)	Distance (Å) [b)]	Atom No. [b)]
1	0.00	23.26	H38-H68
2	0.09	23.56	H32-H68
3	0.09	23.56	H32-H68
4	1.91	26.62	H33-H68
5	2.30	25.86	H33-H68
6	2.17	23.41	H40-H68
7	2.54	23.45	H40-H68
8	2.62	25.49	H33-H68
9	2.83	23.98	H34-H68
10	2.68	24.77	H39-H68
11	2.53	25.66	H33-H68
12	2.79	23.38	H40-H68
13	3.22	23.97	H34-H68
14	3.17	24.62	H39-H68
Average		24.40	

a) "No." corresponds to the energy order in the MMFF94s.
b) Distance between the most distant protons in the molecule.

Supplementary Table 3. Relationship between the **UC13** concentration and the gelation time.*

Concentration (mM)	t_{start} (min)	t_{comp} (min)
3.2	15 ± 0.5	24 ± 0.6
3.0	26 ± 4.5	57 ± 7.7
2.8	97 ± 7.5	155 ± 8.3
2.4	194 ± 38	390 ± 38
2.2	311 ± 67	604 ± 117
2.0	461 ± 173	735 ± 282
1.8	750 ± 205	1,077 ± 248

*All runs were performed under the same conditions.
*For each concentration, the average and standard deviation calculated from ten samples are shown.

Supplementary References

1. Komiyama, T. *et al.* Effect of alkyl chain length of *N*-alkyl-*N'*-(2-benzylphenyl)ureas on gelation. *Chem. Asian J.* **16**, 1750–1755 (2021).
2. Ando, T., Uchihashi, T. & Fukuma, T. High-speed Atomic Force Microscopy for Nano-Visualization of Dynamic Biomolecular Processes. *Prog. Surf. Sci.* **83**, 337–437 (2008).
3. Ishikawa, M., Sugimoto, T., Kikuta, M., Ishiko, E. & Kono, M. Pure Ionic Liquid Electrolytes Compatible with a Graphitized Carbon Negative Electrode in Rechargeable Lithium-ion Batteries. *J. Power Sources* **162**, 658–662 (2006).
4. Seki, S. *et al.* Comprehensive Refractive Index Property for Room-Temperature Ionic Liquids. *J. Chem. Eng. Data* **57**, 2211–2216 (2012).
5. Goto, Y. & Osawa, E. Corner Flapping: A Simple and Fast Algorithm for Exhaustive Generation of Ring Conformations. *J. Am. Chem. Soc.* **111**, 8950–8951 (1989).
6. Gotō, H. & Ōsawa, E. An Efficient Algorithm for Searching Low-energy Conformers of Cyclic and Acyclic Molecules. *J. Chem. Soc., Perkin Trans. 2* 187–198 (1993).
7. Goto, H., Obata, S., Nakayama, N. & Ohta, K. CONFLEX 9 (CONFLEX Corporation, Tokyo, Japan, 2021).
8. Halgren, T. A. Merck Molecular Force Field. I. Basis, Form, Scope, Parameterization, and Performance of MMFF94. *J. Comput. Chem.* **17**, 490–519 (1996).
9. Halgren, T. A. Merck Molecular Force Field. II. MMFF94 van der Waals and Electrostatic Parameters for Intermolecular Interactions. *J. Comput. Chem.* **17**, 520–552 (1996).
10. Halgren, T. A. Merck Molecular Force Field. III. Molecular Geometries and Vibrational Frequencies for MMFF94. *J. Comput. Chem.* **17**, 553–586 (1996).
11. Halgren, T. A. & Nachbar, R. B. Merck Molecular Force Field. IV. Conformational Energies and Geometries for MMFF94. *J. Comput. Chem.* **17**, 587–615 (1996).
12. Halgren, T. A. Merck Molecular Force Field. V. Extension of MMFF94 Using Experimental Data, Additional Computational Data, and Empirical Rules. *J. Comput. Chem.* **17**, 616–641 (1996).
13. Halgren, T. A. MMFF VI. MMFF94s Option for Energy Minimization Studies. *J. Comput. Chem.* **20**, 720–729 (1999).
14. Halgren, T. A. MMFF VII. Characterization of MMFF94, MMFF94s, and Other Widely Available Force Fields for Conformational Energies and for Intermolecular-interaction Energies and Geometries. *J. Comput. Chem.* **20**, 730–748 (1999).
15. Frisch, M. *et al.* Gaussian 16, Revision C.01 (Gaussian, Inc., Wallingford CT, 2016).
16. Grimme, S., Antony, J., Ehrlich, S. & Krieg, H. A consistent and accurate ab initio parameterization of density functional dispersion correction (DFT-D) for the 94 elements H-Pu. *J. Chem. Phys.* **132**, 154104 (2010).
17. http://www.spring8.or.jp/wkg/BL19B2/instrument/lang-en/INS-0000000300/instrument_summary_view
18. S. Kawaguchi., M. *et al. Rev. Sci. Instrum.* **88**, 085111 (2017).
19. Gillespie, D. T. Exact Stochastic Simulation of Coupled Chemical Reactions. *J. Phys. Chem.* **81**, 2340–2361 (1977).

20. Gillespie, D. T. Concerning the Validity of the Stochastic Approach to Chemical Kinetics. *J. Stat. Phys.* **16**, 311–318 (1977).
21. Gillespie, D. T. A General Method for Numerically Simulating the Stochastic Time Evolution of Coupled Chemical Reactions. *J. Comput. Phys.* **22**, 403–434 (1976).
