# Supplementary material for: Molecular-level insights into the supramolecular gelation mechanism of urea derivative
Source: Nat Commun. 2025 Apr 22;16:3758. doi: 10.1038/s41467-025-59032-6 (PMC12015314; doi:10.1038/s41467-025-59032-6)
Supplement: Supplementary file 2 — Description of Addtional Supplementary Files [file 41467_2025_59032_MOESM2_ESM.pdf]

Description of Additional Supplementary Files

Supplementary Movie 1 - HS-AFM movie showing the fibrillation process of **UC13** in DMSO solution.

Supplementary Movie 2 - HS-AFM movie showing the fibrillation of **UC13** in EMI-Tf_2N solution.

Supplementary Movie 3 - HS-AFM movie showing the first stage of the fibrillation process of **UC13**.

Supplementary Movie 4 - HS-AFM movie showing the second stage of the fibrillation process of **UC13**.

Supplementary Movie 5 - HS-AFM movie showing the third stage of the fibrillation process of **UC13**.

Supplementary Movie 6 - HS-AFM movie capturing the elongation process of the thick fiber.

Supplementary Movie 7 - Simulation by the block-stacking model.

Supplementary Movie 8 - Gelation process in a set of vials.
