# Supplementary figures and images for: Molecular-level insights into the supramolecular gelation mechanism of urea derivative
Source: Nat Commun. 2025 Apr 22;16:3758. doi: 10.1038/s41467-025-59032-6 (PMC12015314; doi:10.1038/s41467-025-59032-6)

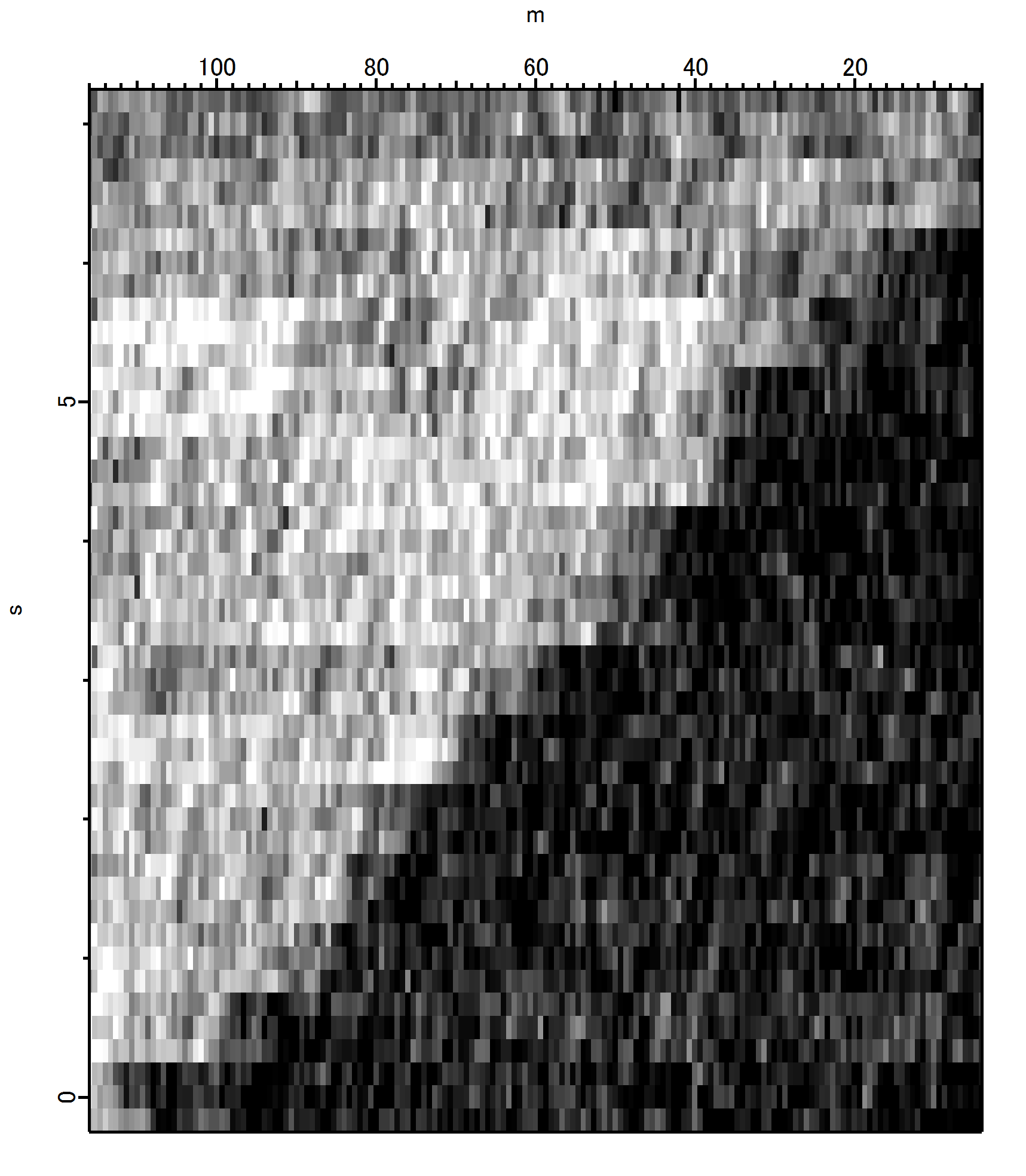

Supplement: Supplementary file 12 — Source Data [file 41467_2025_59032_MOESM12_ESM.zip › Source data/Source Data Fig.4 HS-AFM/Fig4b Kymograph.png]

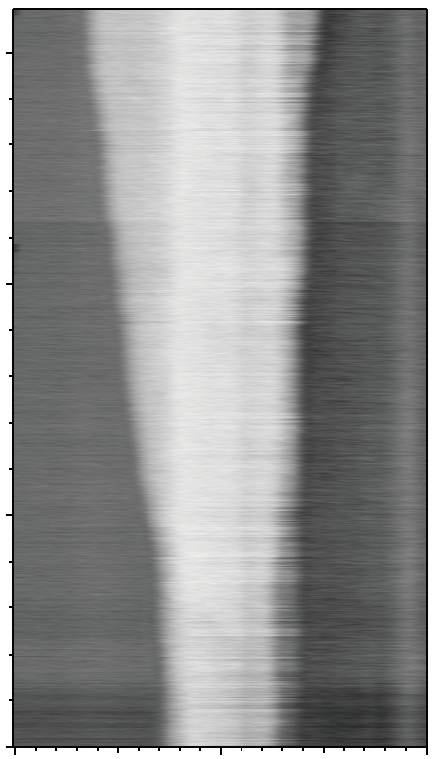

Supplement: Supplementary file 12 — Source Data [file 41467_2025_59032_MOESM12_ESM.zip › Source data/Source Data Fig.5 HS-AFM/Fig5c.png]
